# Approche d’autonomisation d'une communauté africaine dans le diagnostic de soins de santé de deux pays: la Guinée Conakry et le Congo Brazzaville

**DOI:** 10.11604/pamj.2017.28.276.14098

**Published:** 2017-11-28

**Authors:** Gildas Vieira, Robert Courtois, Emmanuel Rusch

**Affiliations:** 1 Fédération Régionale des Acteurs en Promotion de la Santé (FRAPS), Tours, France; 2 CHRU de Tours, Département de Santé Publique et d’Information Médicale, Equipe Emergente ‘Education Ethique Santé’, Tours Cedex 9, France; 3 Université François Rabelais de Tours, PRES Centre-Val de Loire Université, Département de Psychologie, ‘Psychologie des Ages de la Vie et Adaptation’, Tours Cedex, France; 4 CHRU de Tours, Clinique Psychiatrique Universitaire, Tours Cedex 9, France

**Keywords:** Santé communautaire, inégalités sociales en santé, Africains sub-saharien, empowerment, Community health, social inequalities in health, Sub-Saharan Africans, empowerment

## Abstract

**Introduction:**

Les populations d'Afrique subsaharienne, après immigration en France, gardent souvent des modes de vie similaires par communautés, d'autant que les politiques de logements favorisent leurs regroupements dans des quartiers prioritaires. Réfléchir aux enjeux de la promotion de la santé, nécessite d'explorer les politiques de santé des pays d'origine.

**Méthodes:**

Pour cela, nous avons (i) impulsé un travail de réflexion avec un groupe de 16 personnes résidentes en France qui ont été soutenues dans une démarche de renforcement d'empowerment sur la santé communautaire afin d'appréhender les leviers et freins à la santé de leur pays d'origine. Nous avons également (ii) recueilli des données de la littérature avant d'entreprendre plusieurs voyages en Guinée et au Congo afin de confronter ces données à celles du pays.

**Résultats:**

Le résultat concernant la promotion de la santé de ces pays a permis de recenser des mesures à mettre en place. Parmi elles, la facilitation de la santé communautaire en se basant sur des expériences réussies avec la perspective, pour les migrants, de transférabilité en France. Ces mesures impliquent la mobilisation des acteurs institutionnels et des populations dans des démarches éducatives pour un changement de comportement.

**Conclusion:**

Le diagnostic « territorial » permet d'une part de souligner l'importance de l'environnement de santé du pays d'origine sur les comportements adoptés par la suite, d'autre part d'éclairer les solutions pour la promotion de la santé de ces communautés africaines en France.

## Abstract (en)

**Background:**

After immigration to France, the populations of Sub-Saharan Africa have often maintained their traditional lifestyles, this is why housing policies have been promoting their clustering in priority neighborhoods. Discussing issues about health promotion, requires to investigate health policies in their countries of origin.

**Method:**

For this, we (i) organized brainstorming sessions with a group of 16 persons resident in France who were involved in a process of empowerment strengthening of community health programs in order to make them understood the incentives and the obstacles in health care in their countries of origin. We also (ii) collected literature data prior to undertake several trips to Guinea and Congo, in order to compare literature data with those of these countries.

**Result:**

The result concerning health promotion in these countries allowed the identification of measures to be put in place. Among them, the facilitation of accessing to community health programs, basing on successful experiences, with the prospect of transferring them to France for migrants. These measures are based on the involvement of the institutional actors and of the populations in educational approaches to health behavior change.

**Conclusion:**

"Territorial" diagnosis allows to emphasize the importance of the influence of health environment in the country of origin on subsequent behaviours. Moreover, it allows to highlight solutions that can promote harmonization of African community health in France.

## Introduction

Les Guinéens (République de Guinée Conakry) et les Congolais (République du Congo) sont issus de grands pays d'Afrique Centrale et de l'Ouest (Afrique subsaharienne) en voie de développement. Leurs préoccupations de santé sont liées à des conditions de vie souvent difficiles. Les besoins fondamentaux nutritionnels et sanitaires ne sont pas satisfaits pour la plus grande partie de la population. Cela se traduit notamment par un manque de matériel médical, et de formation continue des professionnels [1-3]. Les Guinéens et Congolais doivent prendre en compte les tensions entre la modernité et des aspects traditionnels (croyances, recours aux guérisseurs et aux plantes), sans oublier l'importance du fait religieux (religion à dominante musulmane pour la Guinée Conakry et à dominante catholique pour le Congo) [4]. Lorsqu'ils sont en France, les africains doivent parvenir à faire coexister des attitudes et comportements appropriés au contexte de vie d'origine et celui du pays d'accueil (problématiques culturelles avec des processus syncrétiques et d'acculturation). Afin d´améliorer efficacement leur santé, il est nécessaire d'établir une stratégie de promotion de la santé à partir de leurs besoins. Nous devions prendre en compte les représentations socioculturelles, les contextes géographiques, politiques, socio-économiques, le niveau d'éducation, les systèmes de santé, mais aussi la capacité d'adaptation aux conditions de vie dans le pays d'accueil [5-7]. Cette étude a pour objectif (i) d'apporter un éclairage sur les enjeux de santé en Guinée et au Congo (ii) d'établir un diagnostic qui pourra servir de base à une stratégie d'amélioration des conditions de santé dans les pays d´origine mais aussi en France.

## Méthodes

**Participants:** Dans une démarche d'empowerment (Annexe 1) sur la santé communautaire, nous avons constitué un groupe de 16 migrants originaires d'Afrique, essentiellement issus de Conakry (capitale de la Guinée) ou de Pointe-Noire (seconde ville de la République du Congo et « capitale économique ») et résidant en France, à Blois (Département du Loir-et-Cher, région Centre-Val de Loire). Les personnes ayant réalisées les différentes étapes du diagnostic de santé en France et dans leur pays d'origine étaient aussi bien des habitants des quartiers prioritaires que des professionnels de santé. Cette démarche a été soutenue par la municipalité de Blois.

**Matériel:** Nous nous étions appuyés sur trois types d'outils: (i) un questionnaire construit en séance en France avec le groupe; (ii) des entretiens semi-structurés d'environ 1h à destination des professionnels guinéens et congolais, habitants et personnels du Ministère de la Santé et de la formation professionnelle; (iii) une revue de littératures d'actions de promotion de la santé réussies, effectuées dans les pays d'Afrique subsaharienne. Le contenu des questionnaires utilisés, adapté culturellement, avait pour but d'identifier les acteurs, la population ciblée, ses besoins, les freins et pistes d'actions visant l'amélioration de la santé globale de cette population. En Guinée et au Congo, plusieurs visites et entretiens semi-directifs avec les populations civiles, des politiques et des professionnels de santé ont été réalisé afin d'établir un diagnostic de la situation. Ils étaient basés sur la matrice SWOT (Annexe 1) permettant d'obtenir une vision synthétique d'une situation en présentant les forces et les faiblesses ainsi que les opportunités et les menaces potentielles [8].

**Procédure:** Le is, en focus group (Annexe 1), avec une évaluation de fin d'étude. Les liens avec les représentants Guinéens ont été facilités par un partenariat avec le Ministère de la Santé et de la Formation Technique, ceux avec les Congolais par une convention avec une fondation «Terre nouvelle». Cette démarche sous forme de diagnostic et de partenariat a permis de comprendre l'environnement sanitaire de la santé, social et politique de la Guinée et du Congo, en favorisant un repérage des problèmes de santé que rencontrent les populations, conformément à notre objectif. Nous avons également pu relever des expériences réussies en promotion de la santé dans ces deux pays, permettant d´identifier des pistes d'interventions probantes, notamment en santé communautaire (transposables en France pour ces populations). Dans ce travail nous avons abordé aussi bien les notions de santé publique que de santé communautaire visant le même objectif, c'est-à-dire l'amélioration de la santé des populations. Pour l'atteinte de cet objectif, dans le cadre de la santé publique nous avons eu recours à un processus qu'on pourrait qualifier de technocratique en travaillant avec les politiques, tandis que sur la partie santé communautaire nous avons mis en place un processus participatif alliant focus groupe et conférences. Les actions et interventions en promotion de la santé reposent donc sur la juxtaposition de plusieurs stratégies. La participation communautaire, l'intersectorialité, mais aussi l'empowerment communautaire, ont permis d'accroître la mobilisation et donc l'engagement des différents acteurs. Proposer des formations de professionnels demeure une piste importante à explorer dans une vraie perspective de promotion de la santé. La création d'un Institut de Santé Publique en Guinée et au Congo pourrait constituer une instance de coordination des actions en santé publique et favoriser la formation initiale et continue des acteurs de terrain. Les croyances interculturelles (les représentations qui sous-tendent nos choix) sont à intégrer dans nos processus d'empowerment et de recherche-action afin d'améliorer l'enseignement en santé à destination des populations d'Afrique subsaharienne. Un sujet en marge du diagnostic, qui accompagnait cependant notre réflexion dans ces enjeux de santé pour les populations africaines en France œuvre pour le soutien aux publics précaires. Un comité de pilotage composé de professionnels de santé, de responsables associatifs et de chercheurs, a été mis en place en France afin de garantir le suivi du projet et la validation des outils mis en place. Il s'agissait de travailler sur une démarche communautaire en santé pour favoriser l'empowerment en santé du groupe [9] et recueillir leurs besoins en France. Après évaluation des séances, la nécessité d´aller dans le pays d´origine s´est imposée afin de travailler sur un temps de diagnostic santé [9]. Ce diagnostic a nécessité deux voyages en Guinée et deux autres au Congo avec une délégation restreinte du groupe. Les interventions n'ont été envisagées qu'après avoir pris contact avec les Ministères de la santé guinéen et congolais. L'analyse des déterminants de santé s'est faite en s'appuyant sur les cinq axes de la charte d'Ottawa (Annexe 1) [10] nécessaires à la mise en place d'une stratégie de promotion de la santé visant à favoriser un travail en réseau en s'inscrivant dans une démarche participative groupe des 16 migrants a été réuni une fois par mois sur dix mo des professionnels et habitants du territoire et à aider les populations à exercer un plus grand contrôle sur leur santé. D'un point de vue éthique, nous avons veillé: (i) au respect de l'anonymat; (ii) à la présentation des différents partenaires et du projet; (iii) à la demande expresse à l'ensemble des participants de leur accord pour être interviewé sur la base du questionnaire et (iv) à l'autorisation d'utiliser les éléments de l'échange afin de produire le diagnostic.

**Analyse de données:** Les données issues de l'étude diagnostic en Guinée et au Congo ont été analysées sous l'angle des axes de la charte d'Ottawa qui constitue une grille de lecture et d'analyse des projets. Ce cadre de réflexion désigne à la fois la nécessité de mesures de protection de la santé qui visent à modifier l'environnement social et politique (axes 1, 2 et 5) et la nécessité de renforcer la capacité individuelle et collective d'agir par ses comportements sur les déterminants de sa santé (axes 3 et 4).

## Résultats

**Diagnostic de santé pour la Guinée:** Concernant la Guinée, les résultats d'un diagnostic de santé ont pu être obtenus après de nombreux échanges avec des relais communautaires, des professionnels, des responsables religieux, des politiques (Ministères des Affaires Sociales, de la Santé, de l'Emploi, de la Formation), mais aussi des citoyens (dont des femmes victimes d'excision et des exciseuses).

**Résultats épidémiologiques issues d´enquêtes et de littérature:** Les indicateurs démographiques et de santé de la Guinée ont mis en évidence un état de santé préoccupant de la population. En 2012, la mortalité infantile était de 67‰. Plusieurs maladies transmissibles sont ciblées comme prioritaires dont le groupe VIH (virus de l´immunodéficience humaine) /SIDA (syndrome d'immunodéficience acquise), tuberculose, paludisme et plus récemment Ebola. La malnutrition chronique était de 34,5% [11]. La consommation tabagique est importante et concerne 57% des habitants de Conakry, dont 9% de femmes [12]. Les informations recueillies dans les établissements hospitaliers publics indiquent comme principales causes de mortalité : le paludisme, les infections respiratoires, les maladies cardiovasculaires, les tumeurs et les complications liées à l'accouchement chez les adultes. On notera également l'augmentation progressive du SIDA, du diabète et de l'hypertension artérielle [13].

**Résultat des rencontres avec les institutionnels:** Au cours du séjour en Guinée, nous avons été reçus par la Coordination Nationale de la riposte contre la fièvre à virus Ebola, qui a mis en place plusieurs mesures pour prévenir la propagation de la maladie, dont la multiplication d'actions de santé communautaire dans les quartiers. Son coordinateur national adjoint, en présence du directeur national adjoint de la santé scolaire et universitaire a résumé les actions principales du système de prévention en Guinée en dégageant trois priorités: 1) la mise en place d'un système de santé communautaire axé essentiellement sur une dynamique d'appropriation et d'implication des populations. Cela a impliqué une amélioration de la communication en termes de nombre de personnes à atteindre en passant par les structures scolaires et par le biais des agents communautaires; 2) le renforcement des capacités en ressources humaines qui passe par l'amélioration des contenus de formation initiale et continue incluant l'approfondissement des aspects de prévention tout au long des études; 3) le renforcement du système de formation des formateurs en place.

**Résultats de projets locaux réussis:** Concernant les pistes d'amélioration possibles, nos recherches nous ont conduits à nous intéresser à plusieurs actions de promotion de la santé en Guinée. Elles sont exploitables par leurs résultats positifs et la possibilité de transférabilité des données dans d'autres pays. Le travail fait sur le virus Ebola par exemple a conduit à de réelles implications des communautés bénéficiaires et le renforcement de capacité des populations dans la gestion de leur santé. La question de la promotion de la santé sexuelle et reproductive des jeunes a donné lieu à des centres d'écoute, de conseil et d'orientation des jeunes (CECOJE) créés en Guinée dès 2001, par l'Association Guinéenne pour le Bien-Être Familial (AGBEF). Depuis cette date, une vingtaine de centres a été créée. L'une des particularités de ces centres est la présence d'un personnel jeune qui peut s'adresser plus aisément aux autres jeunes et rompre leurs réticences. Les prestations sont adaptées aux attentes des jeunes en termes d'horaires de travail avec différents services proposés : prise en charge des infections sexuellement transmissibles, test de grossesse, orientation vers les structures sanitaires [14]. Le gouvernement guinéen, en collaboration avec l'UNICEF, a mis en place en 1997 un projet de réduction de la mortalité maternelle et néonatale au niveau du district sanitaire de Dabola. Ce projet était basé non seulement sur l'amélioration de la qualité des prestations mais aussi sur la promotion de la participation communautaire à travers la mise en place de Mutuelles de santé pour la prise en charge des RIsques liés à la Grossesse et à l'Accouchement (MURIGA). Après évaluation de ce projet pilote, l'approche MURIGA a été retenue comme l'une des stratégies nationales de réduction de la mortalité maternelle et a été développée dans 17 des 33 districts sanitaires de la Guinée. Dans le même esprit le projet PRIMA (Projet de Recherche sur le Partage du Risque-Maladie) de recherche-action a mis en œuvre un modèle de participation communautaire au financement du risque maladie [15]. Enfin une expérience de promotion de la santé sur l'immatériel en Guinée Forestière dans la région de Guéckédou (Annexe 1) nous a semblé intéressante: la réparation de la malédiction générale suite à l'enterrement d'une femme enceinte du groupe ethnique « kissi » (Annexe 1) avec le bébé dans le ventre pendant l'épidémie de la Maladie à Virus Ebola en Guinée. Selon la coutume, une césarienne traditionnelle post-mortem pour prévenir une malédiction généralisée était nécessaire, mais les autorités l'interdisent en raison du risque de contamination pour les opérateurs et la population. Il a fallu convaincre la famille d'enterrer cette femme dans son état et parallèlement d'organiser le rituel de réparation de la double malédiction généralisée [16, 17].

**Proposition d'actions de promotion de santé pour la Guinée:** Au regard des entretiens réalisés, nous pouvions proposer certaines mesures: (i) développement des collaborations avec les écoles; (ii) poursuite des efforts de gratuité des soins, tel que l'Etat l'avait mis en place dans le cadre du programme « Césarienne », en particulier pour les maladies les plus fréquemment rencontrées dans le pays (paludisme, problèmes respiratoires et de diarrhées); (iii) développement des partenariats internationaux pour améliorer la formation des professionnels et des personnes ressources. Plusieurs pistes sont proposées: (a) une formation aux premiers secours et gestes d'urgence, évolution des pratiques, (b) des séminaires d'échanges de pratiques et apports de méthodes nouvelles en soins, prévention et promotion de la santé et (c) un développement de partenariats avec les laboratoires afin de permettre la réduction des coûts des médicaments et l'apport de connaissances nouvelles sur les produits et les prises en charge médicales.

**Diagnostic de santé pour le Congo:** Concernant le Congo, les résultats de ce diagnostic ont pu être obtenus après de nombreux échanges avec des relais communautaires, des professionnels, la population, des responsables religieux, des institutionnels du ministère de la santé et du Conseil national de lutte contre le Sida.

**Résultats épidémiologiques issues d´enquêtes et de littérature:** Notre diagnostic a été effectué à Pointe-Noire qui est située sur la façade atlantique du Congo. La ville représente le poumon économique du Congo par son activité pétrolière et son port en eau profonde. Elle est un débouché naturel d'un axe de communication prépondérant pour l'Afrique Centrale. Les entretiens menés sur place permettent d'éclairer: 1) un système de santé à deux vitesses (les cliniques privées dont le nombre ne cesse de croitre sont réservées à la clientèle la plus aisée et l'hôpital public aux plus pauvres); 2) les Centres de santé intégré (CSI) permettent d'assurer les soins des situations simples notamment le suivi des femmes pendant la grossesse et traitent d'autres problèmes de santé comme le SIDA, les troubles liés à la nutrition, etc. Les médecins généralistes y font des consultations gratuites; 3) des analyses de laboratoire sont payantes; 4) des campagnes de vaccination qui sont parfois assurées par des bénévoles qui ne bénéficient pas toujours de compétences médicales; 5) l'absence ou l'insuffisance de prise en charge des addictions et des troubles mentaux; 6) une démographie médicale insuffisante (un seul neurologue à Pointe-Noire, deux neurochirurgiens seulement dans le pays), dont le déficit va s'accentuer avec l'insuffisante offre de formation.

**Résultat des rencontres avec les institutionnels:** La rencontre avec les agents du ministère de la santé ainsi que la conseillère santé du Président de la république, nous a permis de parfaire notre analyse de terrain. Nous notions notamment l'insuffisance des aides internationales (les mesures de l'UNICEF restent trop ponctuelles, notamment concernant le VIH). Le Congo fait face à différents problèmes sanitaires: (i) la gestion des déchets [18] (santé publique et environnemental); (ii) l'insalubrité (le paludisme représentait 54% des consultations en 2002); (iii) les problèmes de santé non réglés comme les infections respiratoires aiguës (qui représentent 34% de consultations générales, première cause de morbidité), les maladies diarrhéiques (qui représentent la seconde cause de morbidité et qui concernent surtout les enfants), le manque de médicaments; l'absence d'une politique globale de prévention et de promotion de la santé; (iv) les efforts qu'il faudrait faire pour l'amélioration de la santé environnementale.

**Résultats de projets locaux réussis:** Au Congo, nous noterons notamment les expériences réussies menées et coordonnées par l'UNICEF [19]. Les principaux résultats et enseignements tirés de la coopération 2009-2013 avec le pays révèlent le rôle catalytique de l'UNICEF pour la promotion d'un système de protection sociale non contributive. Grâce à des mesures de prévention, à titre d'exemple, 187 villages abritant 102461 personnes ont cessé la défécation à l'air libre dans le cadre de l'assainissement total piloté par la Communauté. Une étude exploratoire de la sécurité routière à Brazzaville et à Pointe-Noire a permis une démarche de promotion de la santé auprès des habitants et des élus [20] avec une évaluation très positive sur les évolutions de comportements. Nous constatons cependant des réalisations importantes de l'État en termes de gratuités de certains actes [21], d'amélioration de certaines structures sanitaires, de renouvellement de matériels dans certains établissements.

**Proposition d'actions de promotion de santé pour le Congo:** Différentes pistes d'actions nous semblaient à envisager pour l'amélioration du système de santé et de prévention. Certaines dépendent de la politique de santé au niveau étatique. Les recommandations ou leviers d'amélioration nous paraissaient devoir être: (i) le développement des collaborations avec les écoles; (ii) la poursuite des efforts de gratuité des soins, en particulier pour les maladies les plus fréquemment rencontrées dans les CSI: le paludisme, les problèmes respiratoires et de diarrhées, etc.; (iii) le développement des partenariats internationaux pour améliorer la formation des professionnels et des personnes ressources; (iv) accroître l'accessibilité à l'offre de soins en établissant une tarification adaptée aux familles et à leurs revenus. Son application aux Centres Hospitaliers permettrait de sauver des vies. Nous avons soulevé des opportunités pour l'amélioration de la santé des Congolais: 1) l'amélioration des conditions de soins des malades internés dans les hôpitaux; 2) le renforcement des effectifs qualifiés et de la formation continue; 3) l'augmentation de l'approvisionnement en médicaments et une meilleure gestion de ceux-ci; 4) la meilleure gestion des déchets et politique d'assainissement à améliorer; 5) les moyens matériels pour le personnel soignant.

## Discussion

**Synthèse du diagnostic santé en termes de méthode et de résultat:** Pour la Guinée, comme le Congo, quelques points majeurs ressortent de notre étude. Il est nécessaire: (i) d'améliorer le circuit de prévention et de promotion de la santé notamment au sein de l'éducation nationale afin de rendre plus efficace le message, et contribuer à l'éducation à la santé de la population en formant les professionnels; (ii) de renforcer les démarches de santé communautaire au sein des CSI qui portent satisfaction (notamment pour la prévention mère-enfant), et accentuer les messages de prévention par voies de presses; (iii) de former des agents de santé communautaires [22] qui pourront appréhender la psychologie et la culture de la population et permettre une forme de prévention par les pairs et une meilleure transmission des messages de santé. Ces référents peuvent être aussi importants en France pour les populations migrantes. Il semble également indispensable d'associer les responsables religieux dans les actions de promotion de la santé [23]. L'analyse des résultats a été guidée par les axes de la Charte d'Ottawa sur la promotion de la santé : (i) axes 3 et 4 communautaires et de développement d'aptitude individuelle et (ii) axes 1-2-5 relatifs aux modifications de l'environnement social et politique.

**Action communautaire et aptitude individuelle:** Dans la première phase (renforcer l'action communautaire et la capacité d'agir par ses comportements sur les déterminants de sa santé) (axes 3 et 4), le groupe de 16 migrants a impulsé le projet de recherche-action dans leur pays d'origine. Conformément aux principes de la Charte d'Ottawa qui souhaite influer sur l'environnement physique et social des personnes, le groupe a souhaité contribuer à mettre en place des actions de solidarité et de promotion de la santé. Nous avons participé à des actions communautaires au sein des pays d'origine. Au Congo, en septembre 2015 a eu lieu une Conférence et des échanges avec des associations de santé communautaire. Les associations partenaires que sont La Fraternité Saint Rosaire et la Fondation Terre Nouvelle, nous ont invités à la restitution de nos travaux, sous forme de conférence sur la promotion de la santé, à destination de 333 professionnels éducatifs et de santé au Congo Brazzaville. Cela a été l'occasion de temps d'échanges et de sensibilisation. Nous avons pu travailler sur l'empowerment des populations et les idées reçues sur les différentes maladies comme le virus Ebola, le VIH, l'excision, le dépistage du cancer du col de l'utérus qui sont les thématiques mises en exergue par le groupe pilote. En Guinée à Conakry, nous sommes intervenus de la même manière en février 2016 auprès de plus de 800 professionnels et étudiants avec les associations locales : la Fondation Binta Anne pour les Enfants et les Femmes Excisées (FONBALE) et l'Organisation non gouvernementale (ONG) Sauvons l'Environnement Guinéen. Ces conférences et rencontres politiques ont permis la restitution des résultats, la mobilisation des acteurs locaux et l´acquisition de connaissances pour modifier les comportements de santé.

**Modification des environnements politique et social:** Dans la seconde phase (modifier l'environnement social et politique) (axes 1, 2 et 5 de la charte d'Ottawa), des rencontres avec les acteurs politiques du pays ont été faites et vont se poursuivre, à la fois pour compléter le diagnostic, mais surtout tenter de modifier les politiques de soutien à des actions susceptibles d'amélioration du contexte environnemental en agissant sur les déterminants de santé (conditions de vie, logement, transport, éducation, etc). En Guinée, des échanges ont eu lieu avec le Ministère de la Santé. Nous avions déjà eu la visite de son Secrétaire Général, à Blois en avril 2015. Il nous avait éclairer sur les mécanismes de fonctionnement du système de santé de ce pays et ses limites qui sont liés principalement à l'absence de cohérence entre le regard politique et le terrain fortement impacté par la pauvreté, l'absence ou la vétusté de matériels de soins. Ce dernier avait évoqué les besoins de formation initiale et continue du corps médical et paramédical, la corruption et l'avance difficile d'argent pour des soins d'urgence impactant une population en précarité. Nous avons eu également de nombreux échanges avec les Ministères de l'enseignement supérieur et celui de l'emploi, de l'enseignement technique et de la formation avec qui nous avons signé une convention de partenariat.

Au Congo, nous avons rencontré la conseillère à la santé du président de la république. Nos discussions ont porté sur la manière dont le Congo développait les actions de promotion de la santé notamment sur le volet SIDA, avec l'appui des organismes internationaux dont l'Organisation Mondiale de la Santé (OMS). Ces différentes rencontres ont permis de tisser des liens avec les associations africaines partenaires que nous avons invitées en France. L´utilité d'un Institut de santé publique comme instance de coordination et de soutien des actions et acteurs de terrain est apparue de plus en plus évidente. L´institut permettrait une meilleure coordination des politiques sanitaires, économiques et sociales pour une plus grande équité en santé. C'est précisément ce plaidoyer que nous inscrivons à l'ordre du jour de nos rencontres avec les responsables politiques et ministres de différents secteurs. La Charte d'Ottawa évoque de promouvoir des politiques publiques saines; il s'agit de politiques sociales, économiques, éducatives, des politiques de l'emploi et des loisirs, de l'environnement, de l'urbanisme et de l'habitat. C'est une interpellation directe de tous les décideurs sur leur responsabilité en matière de santé. Notre action a œuvré à développer des politiques et des environnements favorables à la santé en travaillant entre autres avec la Fondation DIENG qui gère les universités Ahmadou Dieng (UAD). Un partenariat avec ce dernier a permis des échanges avec les étudiants et de favoriser l'ouverture d'un cursus universitaire en Santé publique. Nous avons également mis en route un partenariat entre l'hôpital de Blois en France et le Centre Hospitalier Universitaire Ignace Deen de Conakry en Guinée et les universités (UAD) afin de favoriser l'apport de connaissance et la formation en santé publique. Ces partenariats répondent à l'axe 5 de réorientation des services de santé.

## Conclusion

Cette démarche sous forme de diagnostic et de partenariat a permis de comprendre l'environnement sanitaire de la santé, social et politique de la Guinée et du Congo, en favorisant un repérage des problèmes de santé que rencontrent les populations, conformément à notre objectif. Nous avons également pu relever des expériences réussies en promotion de la santé dans ces deux pays, permettant d´identifier des pistes d'interventions probantes, notamment en santé communautaire (transposables en France pour ces populations). Dans ce travail nous avons abordé aussi bien les notions de santé publique que de santé communautaire visant le même objectif, c'est-à-dire l'amélioration de la santé des populations. Pour l'atteinte de cet objectif, dans le cadre de la santé publique nous avons eu recours à un processus qu'on pourrait qualifier de technocratique en travaillant avec les politiques, tandis que sur la partie santé communautaire nous avons mis en place un processus participatif alliant focus groupe et conférences. Les actions et interventions en promotion de la santé reposent donc sur la juxtaposition de plusieurs stratégies. La participation communautaire, l'intersectorialité, mais aussi l'empowerment communautaire, ont permis d'accroître la mobilisation et donc l'engagement des différents acteurs. Proposer des formations de professionnels demeure une piste importante à explorer dans une vraie perspective de promotion de la santé. La création d'un Institut de Santé Publique en Guinée et au Congo pourrait constituer une instance de coordination des actions en santé publique et favoriser la formation initiale et continue des acteurs de terrain. Les croyances interculturelles (les représentations qui sous-tendent nos choix) sont à intégrer dans nos processus d'empowerment et de recherche-action afin d'améliorer l'enseignement en santé à destination des populations d'Afrique subsaharienne [24]. Un sujet en marge du diagnostic, qui accompagnait cependant notre réflexion dans ces enjeux de santé pour les populations africaines en France.

### Etat des connaissances actuelles sur le sujet

- La santé communautaire comme outil de promotion de la santé utilisée en Afrique subsaharienne notamment pour véhiculer les messages de prévention lors d'épidémies et de campagnes de vaccina-tion;
- Les diagnostics dans ces pays notamment ceux de l'UNICEF permettent d'illustrer la situation en santé publique en Afrique et d'apporter des réponses appropriées avec les gouvernants.

### Contribution de notre étude à la connaissance

- Une méthodologie originale de diagnostic sur 2 pays (Afrique de l'Ouest et Central) par les paires (personnes originaires d'Afrique subsaharienne) initiés par un travail préalable en France de 10 séances de santé Communautaire;
- Une démarche de diagnostic communautaire en santé identifiant des projets réussis transpo-sables dans d'autres pays d'Afrique mais aussi vers la France. Des données probantes ou promet-teuses;
- Une démarche d'empowerment en lien avec les gouvernants et politiques de Guinée et du Congo (associés à la réflexion en France et en Afrique) et la population aboutissant à des solutions d'importance comme la mise en place d'instituts de santé publique, processus dans lequel nous avons su les impliquer.

## Conflits d’intérêts

Les auteurs ne déclarent aucun conflit d'intérêts.

## Annexe

**Annexe 1**: glossaire
